# Supplementary material for: Comparison of Foraging Interactive D-prime and Angular Indication Measurement Stereo with different methods to assess stereopsis
Source: PLoS One. 2024 Jun 7;19(6):e0305036. doi: 10.1371/journal.pone.0305036 (PMC11161055; doi:10.1371/journal.pone.0305036)
Supplement: Attachment — Submitted filename: Response to Reviewers.docx [file pone.0305036.s001.docx]

Northeastern University

105-107 Forsyth St,

Boston, MA, 02115

neupanesonisha@gmail.com

The Academic Editor

Investigative Ophthalmology & Vision Science

Feb 22, 2024

Dear Editor,

We thank you and the reviewers for your time and comments on our manuscript. In this letter, we provide a detailed response to the points raised by the reviewers.

We have highlighted the reviewers’ comments in bold, our responses are in blue plain text. We have also included the original and modified text where applicable and we have used italics for the lines from the manuscript.

We have revised the funding statement and conflict of interest as follows:

**Funding Statement**

This project was supported by National Institutes of Health (www.nih.gov)
(grant R01 EY029713 to PJB). There was no additional external funding received for this study. The funders had no role in study design, data collection and analysis, decision to publish, or preparation of the manuscript.

**Competing Interests:**

I have read the journal's policy and the authors of this manuscript have the following competing interests:

FInD and AIM technologies are disclosed as provisional patented (AIM) and pending patent (FInD) and held by Northeastern University, Boston, USA.

FInD title: Method for visual function assessment; Application PCT/US2021/049250

AIM title: Self-administered adaptive vision screening test using angular indication.

Application PCT/US2023/012959.

JS and PJB are founders and shareholders of PerZeption Inc, which has an exclusive license agreement for FInD and AIM with Northeastern University. SN declares that she has no conflict of interest.

This does not alter our adherence to PLOS ONE policies on sharing data and materials.

Yours sincerely,

Sonisha Neupane

**Journal Requirements:**

**When submitting your revision, we need you to address these additional requirements.**

**1. Please ensure that your manuscript meets PLOS ONE's style requirements, including those for file naming. The PLOS ONE style templates can be found at**

[**https://journals**](https://journals/)**.plos.org/plosone/s/file?id=wjVg/PLOSOne_formatting_sample_main_body.pdf and**

[**https://journals.plos.org/plosone/s/file?id=ba62/PLOSOne_formatting_sample_title_authors_affiliations.pdf**](https://nam12.safelinks.protection.outlook.com/?url=https%3A%2F%2Fjournals.plos.org%2Fplosone%2Fs%2Ffile%3Fid%3Dba62%2FPLOSOne_formatting_sample_title_authors_affiliations.pdf&data=05%7C02%7Cs.neupane%40northeastern.edu%7C30213133a923410deb2c08dc109cb879%7Ca8eec281aaa34daeac9b9a398b9215e7%7C0%7C0%7C638403512846382487%7CUnknown%7CTWFpbGZsb3d8eyJWIjoiMC4wLjAwMDAiLCJQIjoiV2luMzIiLCJBTiI6Ik1haWwiLCJXVCI6Mn0%3D%7C3000%7C%7C%7C&sdata=FRH7Ng%2FK5azwcngZuaSAH1ZpqyqeJqWWwB6eCG%2F%2Bn3Y%3D&reserved=0)**.**

We have edited the manuscript according to PLOS ONE’s style requirements.

**2. Did you know that depositing data in a repository is associated with up to a 25% citation advantage (https://doi.org/10.1371/journal.pone.0230416)? If you’ve not already done so, consider depositing your raw data in a repository to ensure your work is read, appreciated and cited by the largest possible audience. You’ll also earn an Accessible Data icon on your published paper if you deposit your data in any participating repository** ([https://plos.org/open-science/open-data/#accessible-data](https://nam12.safelinks.protection.outlook.com/?url=https%3A%2F%2Fplos.org%2Fopen-science%2Fopen-data%2F%23accessible-data&data=05%7C02%7Cs.neupane%40northeastern.edu%7C30213133a923410deb2c08dc109cb879%7Ca8eec281aaa34daeac9b9a398b9215e7%7C0%7C0%7C638403512846539920%7CUnknown%7CTWFpbGZsb3d8eyJWIjoiMC4wLjAwMDAiLCJQIjoiV2luMzIiLCJBTiI6Ik1haWwiLCJXVCI6Mn0%3D%7C3000%7C%7C%7C&sdata=oIodt72GrBsOWuDeTlb%2BM%2B0iMsl9QnZvhpXF%2FwOZ9%2F4%3D&reserved=0)).

We have deposited the data and the MATLAB code in the Zenodo repository. The DOI for our data and code is 10.5281/zenodo.10688863.

**3. Thank you for stating in your Funding Statement:**

**“This project was supported by National Institutes of Health (www.nih.gov)
(grant R01 EY029713 to PJB).”**

**Please provide an amended statement that declares *all* the funding or sources of support (whether external or internal to your organization) received during this study, as detailed online in our guide for authors at http://journals.plos.org/plosone/s/submit-now.  Please also include the statement “There was no additional external funding received for this study.” in your updated Funding Statement.**

**Please include your amended Funding Statement within your cover letter. We will change the online submission form on your behalf.**

This project was supported by National Institutes of Health (www.nih.gov)
(grant R01 EY029713 to PJB). **There was no additional external funding received for this study.** The funders had no role in study design, data collection and analysis, decision to publish, or preparation of the manuscript.

**4. We note that you have a patent relating to material pertinent to this article. Please provide an amended statement of Competing Interests to declare this patent (with details including name and number), along with any other relevant declarations relating to employment, consultancy, patents, products in development or modified products etc. Please confirm that this does not alter your adherence to all PLOS ONE policies on sharing data and materials, as detailed online in our guide for authors http://journals.plos.org/plosone/s/competing-interests by including the following statement: "This does not alter our adherence to  PLOS ONE policies on sharing data and materials.” If there are restrictions on sharing of data and/or materials, please state these. Please note that we cannot proceed with consideration of your article until this information has been declared.**

**This information should be included in your cover letter; we will change the online submission form on your behalf.**

I have read the journal's policy and the authors of this manuscript have the following competing interests:

FInD and AIM technologies are disclosed as provisional patented (AIM) and pending

patent (FInD) and held by Northeastern University, Boston, USA.

FInD title: Method for visual function assessment; Application PCT/US2021/049250

AIM title: Self-administered adaptive vision screening test using angular indication;

Application PCT/US2023/012959.

JS and PJB are founders and shareholders of PerZeption Inc, which has an exclusive

license agreement for FInD and AIM with Northeastern University. SN declares that she has no conflict of interest. **This does not alter our adherence to PLOS ONE policies on sharing data and materials.**

**5. When completing the data availability statement of the submission form, you indicated that you will make your data available on acceptance. We strongly recommend all authors decide on a data sharing plan before acceptance, as the process can be lengthy and hold up publication timelines. Please note that, though access restrictions are acceptable now, your entire data will need to be made freely accessible if your manuscript is accepted for publication. This policy applies to all data except where public deposition would breach compliance with the protocol approved by your research ethics board. If you are unable to adhere to our open data policy, please kindly revise your statement to explain your reasoning and we will seek the editor's input on an exemption. Please be assured that, once you have provided your new statement, the assessment of your exemption will not hold up the peer review process.**

We have deposited the data and the MATLAB code in the Zenodo repository. The DOI for our data and code is the 10.5281/zenodo.10688863.

**6. We note you have included a table to which you do not refer in the text of your manuscript. Please ensure that you refer to Table1 & 2 in your text; if accepted, production will need this reference to link the reader to the Table.**

We have referred the Table 1 and 2 in the text.

XXXXXXXXXXXXXXXXXXXXXXXXXXXXXXXXXXXXXXXXXXXXXXXXXXXXXXXXXXXXXXXXXXXXXXXXXXXXXXX

[Note: HTML markup is below. Please do not edit.]

Reviewers' comments:

Reviewer's Responses to Questions

Comments to the Author

1. Is the manuscript technically sound, and do the data support the conclusions?

The manuscript must describe a technically sound piece of scientific research with data that supports the conclusions. Experiments must have been conducted rigorously, with appropriate controls, replication, and sample sizes. The conclusions must be drawn appropriately based on the data presented.

Reviewer #1: Yes

Reviewer #2: Partly

2. Has the statistical analysis been performed appropriately and rigorously?

Reviewer #1: No

Reviewer #2: Yes

3. Have the authors made all data underlying the findings in their manuscript fully available?

The [PLOS Data policy](http://www.plosone.org/static/policies.action#sharing) requires authors to make all data underlying the findings described in their manuscript fully available without restriction, with rare exception (please refer to the Data Availability Statement in the manuscript PDF file). The data should be provided as part of the manuscript or its supporting information, or deposited to a public repository. For example, in addition to summary statistics, the data points behind means, medians and variance measures should be available. If there are restrictions on publicly sharing data—e.g. participant privacy or use of data from a third party—those must be specified.

Reviewer #1: Yes

Reviewer #2: No

4. Is the manuscript presented in an intelligible fashion and written in standard English?

PLOS ONE does not copyedit accepted manuscripts, so the language in submitted articles must be clear, correct, and unambiguous. Any typographical or grammatical errors should be corrected at revision, so please note any specific errors here.

Reviewer #1: Yes

Reviewer #2: Yes

XXXXXXXXXXXXXXXXXXXXXXXXXXXXXXXXXXXXXXXXXXXXXXXXXXXXXXXXXXXXXXXXXXXXXXXXXXXXXXX

**5. Review Comments to the Author

Please use the space provided to explain your answers to the questions above. You may also include additional comments for the author, including concerns about dual publication, research ethics, or publication ethics. (Please upload your review as an attachment if it exceeds 20,000 characters)**

**Reviewer #1: General Comments:

The study introduces two novel stereo measurement techniques, AIM and FlnD, and compares them with established tests such as 2AFC, Randot, and Titmus fly test. While the manuscript is technically sound, there needs a major restructuring with clarification and refinement for several points.**

We thank the reviewer for taking the time to evaluate our paper and addressed each of the comments. To address the reviewers request for clarification and to improve the legibility of the revision, we changed the structure of all sections. We hope that the reviewer finds, that due to these changes, the overall legibility and quality of the manuscript has improved.

**Clarity of Stimuli: The description of the FlnD noise stimulus needs further clarification. The term "noise" typically implies random dots without any signal. Consider renaming the target or providing a clear description of what subjects saw and responded to.**

We thank the reviewer for the suggestion and have added the stimulus depth figures and edited the text to make it clear. We have also changed the name from ‘noise’ to ‘dip’ e.g. FInD Dip to avoid confusion.

**Viewing Distance: Ensure that details about the self-administrable tests, such as FlnD, AIM, and 2AFC, include information on how the viewing distance was maintained at 80 cm. Inconsistencies in viewing distance could contribute to data variation.**

We have included the following line in the Method Section.

*Line 124: “A chinrest was used to maintain the viewing distance.”*

**Aim of the study: Lines 137-144 outline the aims of the study but could be clarified. The lines starts out to stating there are 2 aims and explaining 5 aims. Consider explaining the features of FlnD and AIM in the introduction and focusing on the aims and objectives in this section.**

We have edited the text to make it clear that two experiments were conducted to address five research aims.

*Line 93-100:” The two experiments reported here were performed as proof-of-concept studies with five aims: First, we introduce FInD Stereo and its features. Second, we compare estimates of stereoacuity, test duration, and test-retest reliability of FInD with standard 2-AFC methods, and clinically used tests (Randot and Titmus) in stereo-typical and atypical participants. Third, we compare different spatial properties of stereo-inducing stimuli and examine their effect on stereoacuity. Fourth, we introduce AIM Stereo and its features. Fifth, we compare AIM Stereo against the above-mentioned clinical tests using the same outcome measures.”*

**Sections Organization: It is highly recommended that the FlnD and AIM results be combined as a single experiment for better coherence. The readers will be more interested in learning about the two paradigms rather than learning how the AIM came into being and to solve which problems of the FlnD paradigm. The author can discuss why AIM performed better than FlnD at the stereo thresholding in the discussion section.**

The FInD and AIM were done at different times with different set of participants. To reflect this, we have kept them as two experiments, but we have edited the text to make the flow better.

**Stats: Using stats to compare the results between the normal and binocular impaired individuals will lead to false results due to the huge uneven sample size**

We appreciate the comment and acknowledge this limitation of our study accordingly.

*Line 523-524: A limitation of this study is the small number of binocularly impaired participants in the FInD experiment and none in AIM experiment.*

**Specific Line-by-Line Comments:
Line 52: The classic sign for macular degeneration includes macular atrophy, drusens etc and the impact of these is loss of stereopsis. Stating stereopsis as a biomarker for degenerative diseases will be an error.**

We have removed the section stating stereopsis as a biomarker for degenerative disease.

**Line 115: Recheck the reference for the loss of stereoacuity due to refractive error and amblyopia as it refers to a paper related to retinal eccentricity.**

We have updated the reference.

**Line 195-196: Provide consistency in units for the lower and upper bound min and max disparity, considering reporting in degrees.**

We have changed the text to report them in degrees.

*Line 153-156: On the first chart, the disparity range was scaled to span 0.005° (0.3 arcmin) to 0.5° (30 arcmin) (which were also the upper and lower bound min and max disparity) in evenly spaced log steps to cover the broad typical stereoacuity range for binocularly healthy adults.*

**Line 200-204: Clarify how the targets for the ring and noise stimuli were shifted towards opposite side and yet one produces crossed and another produces uncrossed disparities.**

It depends on which side of the disparities the eyes are seeing. If the right side targets are visible to right eye and left side to left eye, it produces the uncrossed disparities whereas if it is the reverse, it provides the crossed disparities.

**Line 275-276: Complete the sentence “Matlab’s anovan and multcompare functions for ANOVA and planned comparisons between tests”.**

We have changed the sentence accordingly.

Line 235-236: *Threshold estimates were analyzed with Matlab’s anovan and multcompare functions for ANOVA and planned comparisons were used to study the variance between tests.*

**Line 290: Address the distribution of data in the statistical analysis section (if the data is normally distributed). Also, 2-way ANOVA was performed but looking at the fig 3, FlnD data, the mean and median are quite apart from each other.**

Some of the outcomes are not normally distributed. For data from Fig 3, we did log transformation to convert them to normally distributed data before performing ANOVA. The log threshold data (Figure 4) was not able to convert to normally distribution after further transformation and we have stated in the manuscript stating so.

*Line 245-247: Log-transformation was applied before using ANOVA test to transform the skewed data to normally distributed data.*

*Line 275-276: The application of the data transformation failed to convert the skewed data to normally distributed data.*

**Line 299: Clarify that whiskers usually represent 1st and 99th percentiles, not outliers.**

We have changed the text to clarify that.

*Line 259-261: Data points show the results for individual observers expressed by a horizontally jittered kernel density, means are depicted in dark squares, boxes indicate the 25-75% interquartile range, whiskers represent 1st and 99th percentiles.*

**Line 306: Explain the rationale for excluding values >3000 arcsec from analysis. Specially when they belong to the binocular impaired group, one would expect the stereo to be quite poor. If the data is skewed because of those subjects, a possibility would be to renumber stereo thresholds >3000 as 3000 and mention the reason for 3000 arcsec as the cutoff.**

The Titmus fly is equal to 3552’’ threshold but has variable disparity intensities between wings and legs. Thus, we have decided 3000’’ as similar criteria for our analysis.

*Line 268-270: Values greater than 3000 arcsec (3.48 log arcsec) were removed from the data analysis, resulting in the removal of 24 thresholds from 3 control participants (total: 608 thresholds) and 24 thresholds for 2 binocularly impaired participants (total: 152).*

**Line 310-312: Discuss the potential implications of the observed variability.**

We have modified the text as follows to make our point clear.

Line 273-275: The binocularly impaired participants had a wide range of stereoacuity in the clinical tests and this was observed with the other tests in Figure 4.

**Line 314: p-value can never be exactly 0.**

We have changed that to p<0.001.

**Line 345: “purple dot” – please keep colours consistent**

We have made the colors consistent and used magenta for binocularly abnormal participants in Figures 3, 4 and 6.

**Line 347: “Each figure contains 1 data point for each participant for Randot” shouldn’t the total number of dots be 20?**

As most of the participants had the same results for Run 1 and 2, their results overlapped.

**Line 406: Provide reasons for not including 2AFC in the comparison; consider combining the first and second experiments.**

The study progressed from the first experiment validating FInD with adaptive and static clinical test and the second part introducing AIM Stereo improving upon the FInD data as proof of concept. We acknowledge the missing comparison with an adaptive method in the future direction section accordingly.

Line 236-239: “The current study introduced AIM Stereo and compared its result with conventional clinical stereovision tests as proof-of-concept. We will compare AIM Stereo to other adaptive techniques, e.g., 2 AFC methods in future studies.”

**Line 457: Clarify the unit "5 X 1.25."**

We have added the *𐩑*  to clarify the units were in degrees (Line 402).

**Line 457: It will be advisable to look at the learning in 2AFC and FlnD?**

We thank the reviewer for the suggestion. We investigated bias using Bland Altman plots and did not find any systematic learning effect during the retest. We address this point accordingly in the discussion:

Line 493-496: We investigated repeatability using Bland Altmann plots and found no systematic learning effect as indicated as bias for FInD Stereo tests, 2-AFCs, or clinical tests for both controls and binocularly-impaired individuals (Figure 6). The same was found for AIM Stereo (Figure 11). AIM Stereo showed less test-retest variability than FInD Stereo.

**Line 489-499: Avoid repetition of introduction points and directly transition to conclusion statements.**

We thank the reviewer for the tip and have removed the repetition accordingly.

**Line 514: AIM was not tested on amblyopes in this manuscript.**

We thank the reviewer for spotting this error and have removed the AIM paradigm to make it clear we only tested amblyopes with FInD method.

**Comments on the Figures:
Fig 3:
The different colours for the 6 tests are not required.**

We have changed Figure 3 accordingly. Now, the control participants are designated by blue and the binocularly impaired participants are designated by magenta colors.

**The horizontal jittering in 2AFC and clinical test seems to be huge. Please maintain uniform jittering.**

The points are jittered based on a kernel density function. Hence, they do not look uniform as the kernel density results differed between tests.

**Figure 4:
The normal can be given a single colour while the binocular impaired can be assigned a different colour and kept that uniform with figure 3. Also, number of subjects are not consistent.**

The normal and binocularly impaired are given different colors and are consistent with previous figure.

We do not have data from one binocular impaired participant for 2AFC Noise and removed other data as the stereoacuity was >3000’’. Otherwise, the number of participants were consistent (19 for normal and 5 for binocular impaired). We clarified these omissions in the manuscript.

*Line 268-270: Values greater than 3000 arcsec (3.48 log arcsec) were removed from the data analysis, resulting in the removal of 24 thresholds from 3 control participants (total: 608 thresholds) and 24 thresholds for 2 binocularly impaired participants (total: 152).*

**Figure 5:
Consider combining with Figure 4 as panel E for easier comparison.**

We have combined the figures accordingly.

**Figure 6:
Unequal number of subjects are presented here.**

We thank the reviewer for the comment. We have changed the figure 5 including axis-scale, which was skewing data and caused data point to overlap.

**The graphs are repeated, for example randot Vs Titmus fly, or Titmus fly vs Randot will be same. Consider keeping only one to avoid confusion.**

We have removed the repetitions (figure 5).

**What additional inferences does the histogram generate?**

The histograms show how the data are distributed for each condition and each test and reveals differences between correlations.

**Are these data points average of the 4 spatial frequency?**

We thank the reviewer for bringing this to our attention. Yes, for FInD and 2-AFC tests, the data points are the average from 4 spatial frequencies because we did not find a significant effect between conditions.

We clarified in the descriptor of this figure 5.

Line 301:” *FInD and 2 AFC data are averaged across spatial frequency conditions.”*

**Consider including the Rsq value**

We have included R^2^.

**Figure 7:
Align captions above the graphs for clarity.**

We have aligned the captions accordingly.

**Figure 11: Please remove repetitions of the graphs and mention the R sq and p values.**

We have removed the repetitions and included Rsq and p values.

XXXXXXXXXXXXXXXXXXXXXXXXXXXXXXXXXXXXXXXXXXXXXXXXXXXXXXXXXXXXXXXXXXXXXXXXXXXXXXX

**Reviewer #2: The study reports the inter-technique comparison and intra-technique repeatability of two new psychophysical measures of stereoacuity, relative to conventional clinical techniques and 2AFC psychophysical techniques. The two new tests were themselves quite repeatable but there was very little agreement between the various tests of stereoacuity. The time taken for the two new tests were shorter than conventional 2AFC techniques but significantly longer than the clinical tests of stereoacuity. These results are discussed in the context of a speed-accuracy trade-off in the assessment of stereoacuity.

The manuscript is easy to read and more or less free of grammatical errors. There are some confusing sentences dispersed all through the manuscript (indicated below in the specific comments) that need to be fixed. The figures are all of very low resolution and the details were not visible upon magnification. This needs to be fixed as well. In addition to these issues, the following issues need to be addressed in the manuscript.**

We are sorry for the inconvenience. The complied submission PDF includes low-resolution preview images due to formatting guidelines of the journal. High resolution figures can be downloaded by clicking the link at the top of each preview page. We have also submitted the figures with better resolution.

**1. While I do not have any concern with the quality of the science pursued in this manuscript, I worry about the utility of these techniques in the future assessment of stereoacuity in the clinic/research settings. This concern is more for the clinical application, and it primarily stems from the time it takes to complete the test, vis-à-vis, the present techniques of Titmus or RanDot stereoacuity. I appreciate that the two new techniques are far more scientifically rigorous, and they overcome several limitations fraught with the present testing paradigms. However, is this a motivation enough for an average clinician to switch over to this technique? These techniques take ~3-times the time to complete than the present techniques and in a busy clinic, this translates into significant chair time for the patient. I fear that the accuracy benefit obtained using this technique may be over-ridden by the time taken to complete the task.
There may be a solution to this though. As indicated in the discussion section of the manuscript (lines 506 – 509), testing only one spatial frequency stimulus or a single disparity sign may reduce the time to complete the task. Given that the target spatial frequency did not have an impact on the stereoacuity anyways, can this not be implemented in the present version of the software and evidence provided that this indeed reduces the time to do the task? I understand that this involves additional data collection and more work for the authors, but this may be a significant step towards the holy-grail of having a test that is both accurate and can be administered quickly.**

Yes, testing only one spatial frequency (FInD) and only one sign reduces the time to do the task. We are now reporting average single chart durations for both FInD (18sec) and AIM (60sec). Therefore, when using only one condition with 2 charts, the duration is in the vicinity of the clinical tests, but with the additional advantages that they a) do not require additional administrator chair time due to the self-administered approach and b) use responses adaptive procedures that build personalized performance models.

**2. The stimuli used in the two new stereoacuity techniques is not clear to me. There are bandpass filtered ring and noise stimuli that the participants had to appreciate depth in. What was the pattern of depth though? I assume that in the ring stimuli, the “ring” appeared in depth depending on the stimulus disparity used but what was seen in the “noise” stimuli? This is not clear from the explanation. Perhaps a figure showing the two stimuli may be useful to include here for helping the readers understand the stimulus.**

We have added the stimulus depth figures and edited the text to make it clear. We have also changed the name from ‘noise’ to ‘dip’ e.g. ‘FInD Dip’ throughout the manuscript to avoid confusion. See figure 1.

**3. Other specific issues…
a. Abstract conclusion: Why only FlnD detected stereo deficits. It is also detected by AIM right? Please rephrase.**

We did not test binocularly impaired participants with AIM, hence we did not include AIM in the statement. This work will be part of a separate study.

**b. Abstract conclusion: The last statement is a rather superficial one. It should be replaced with a statement that describes the advantage/challenges of switching over to the new techniques proposed in this study, vis-a-vis, the existing ones. The new techniques take ~3times longer to complete than the routine clinical tests. What would be the motivation for the clinician to switch over to the new technique should come out clearly here.**

We have changed the abstract to address this point.

**c. Line 78: Add to this list that these tests are meant to be run at a constant viewing distance and are meant for a single IPD - the former limits their ability to check stereopsis at different viewing distances while the latter creates issues with *test accuracy.***

We have added the “assumptions based on testing distance and interpupillary distance and less flexibility on testing distance” as the problems in booklet-based clinical methods in Line 59.

**d. In general, the introduction is way too long (4.5 pages). This can be significantly shortened, without losing information.**

We have significantly shortened the introduction.

**e. Line 123: Expand HMD’s.**

We have replaced HMD’s with Head-mounted displays in the text.

**f. Lines 137 - 144: This section is a bit confusing. The start makes it appear as if there are only two experiments, but the remainder of the manuscript appears like there are five parts to the study. So, add a statement before first to indicate that what ensues is a note on the organization of the manuscript.**

We have edited the text to make it clear there are two experiments with five aims (Line 93-100).

**g. Line 170: I am sure this was taken care of, but a brief mention that there was no leak of information between the red/blue filters to reassure the readers. Also, how was the issue of blur arising from chromatic aberrations of the eye handled in these stimuli?**

We used supra-threshold stimuli whereby cross-talk was not visible.

Our participants wore either no or only low refractive error correction while performing a central fixation task. Hence, we have no reason to think that chromatic aberration influenced the results.

**h. Line 195: Should this be log arc sec?**

They were 0.005° (0.3 arcmin) to 0.5° (30 arcmin) which we have corrected in the text.

*Line 153-156: On the first chart, the disparity range was scaled to span 0.005° (0.3 arcmin) to 0.5° (30 arcmin) (which were also the upper and lower bound min and max disparity) in evenly spaced log steps to cover the broad typical stereoacuity range for binocularly healthy adults.*

**i. Lines 286 – 287: Please include a measure of variance of the data in all measures. Also, please fix the p=0.000 to p<0.001.**

We have changed the ‘p=0.000’ to ‘p<0.001’.

**j. Line 292: Use of Box plots suggests that data were not normally distributed. Was that the case? If so, the results need to be presented as median and IQR.**

Yes, some of the data were not normally distributed. We have used median and IQR log transformed the data where applicable.

**k. Line 517: Be explicit that the new tests are faster, relative to the 2AFC paradigms.**

We have modified the original text as follows:

*Line 466-468:* *There was good agreement between stereoscopic thresholds measured FInD and classic 2AFC paradigms, suggesting that the faster speed of FInD did not come at the loss of accuracy.*

**l. Like the introduction section, the discussion section could also be tightened. In several areas of this section, the results are repeated, and this redundancy can be removed.**

We have tightened the discussion section, which improved the legibility of the manuscript. Last but not least we would like to thank the reviewer for taking the time to evaluate our manuscript.

XXXXXXXXXXXXXXXXXXXXXXXXXXXXXXXXXXXXXXXXXXXXXXXXXXXXXXXXXX

**6. PLOS authors have the option to publish the peer review history of their article. If published, this will include your full peer review and any attached files.

If you choose “no”, your identity will remain anonymous but your review may still be made public.

Do you want your identity to be public for this peer review? For information about this choice, including consent withdrawal, please see our Privacy Policy.**

**Reviewer #1: No**

**Reviewer #2: No**

XXXXXXXXXXXXXXXXXXXXXXXXXXXXXXXXXXXXXXXXXXXXXXXXXXXXXXXXXX
