# Supplementary material for: Comparison of Foraging Interactive D-prime and Angular Indication Measurement Stereo with different methods to assess stereopsis
Source: PLoS One. 2024 Jun 7;19(6):e0305036. doi: 10.1371/journal.pone.0305036 (PMC11161055; doi:10.1371/journal.pone.0305036)
Supplement: Attachment — Submitted filename: Response to Reviewers.docx [file pone.0305036.s002.docx]

Northeastern University

105-107 Forsyth St,

Boston, MA, 02115

neupanesonisha@gmail.com

The Academic Editor

Investigative Ophthalmology & Vision Science

May 20, 2024

Dear Editor,

We thank you and the reviewers for your time and comments on our manuscript. In this letter, we provide a detailed response to the points raised by the reviewers.

We have highlighted the reviewers’ comments in bold, our responses are in blue plain text. We have also included the original and modified text where applicable and we have used italics for the lines from the manuscript.

Yours sincerely,

Sonisha Neupane

**Comments to the Author**

**Reviewer #1**: Really appreciate the effort taken to incorporate the changes into the manuscript.

We thank the reviewer for the appreciation.

The explanation of the aims is still not proper yet. What do the authors mean by two experiments? Was it the development of the FlnD and AIM? The author might want to avoid saying it's two experiments.

Thank you for the suggestion, we originally described the development of the FlnD and AIM as 2 groups of experiments. However, to avoid confusion, we have changed the presentation into Study 1 and 2, one for each novel method, and each with experiments that were completed to investigate stereoacuity with each method. The 2 Studies are differentiated by the main novel method (FInD or AIM) and by different subject pools.

*Line 91-99: The two studies reported here were performed as proof-of-concept studies for 2 novel methods to measure stereoacuity: In Study One, we introduce FInD Stereo and its features. We compare estimates of stereoacuity, test duration, inter-test reliability, and repeatability of FInD with standard 2-AFC methods, and clinically used tests (Randot and Titmus) in stereo-typical and atypical participants. We also use the FInD method to compare different spatial properties of stereo-inducing stimuli and examine their effect on stereoacuity. In Study Two, we introduce AIM Stereo and its features, then we compare AIM Stereo against the above-mentioned clinical tests using the same outcome measures.*

Figure 1 is impressive as it explains clearly, what the subjects would have been able to see. However, that makes me wonder why would the authors wanted to create these two stimuli. Authors might want to add a statement to indicate the pros of the ring and dip stimulus.

We have added a statement in methods to indicate that the ring and dip stimulus investigate different aspects of stereopsis.

*Line 144-148: The ring and dip stimuli investigate different aspects of stereopsis. The ring stimuli consist of sparse contour features, generally referred to as ‘local’ stereopsis, whereas the dip stimuli are defined by dense noise elements, generally referred to as ‘global’ stereopsis. These stimulus types have been used in different populations and with some evidence for separate processing mechanisms.*

Lines 244 to 247 are a part of statistical analysis. Also in that section, it will be better to explain which variables were not normally distributed and that the authors have log-transformed the variable.

We have edited the statistical analysis section to include the following:

*Line 241-248: Duration data were skewed for FInD and Titmus data (Study 1) and AIM data (Study 2) and log-transformation was applied to convert durations to normally distributed data. Threshold estimates were log-transformed to convert the stereo-values to log-stereoacuity. The data for ring scotoma (FInD and 2AFC) and clinical tests (both Study 1 and 2) were still skewed, and further transformation did not convert it to normally distributed data. Hence, Wilcoxon signed rank test and Kruskal-Wallis tests were performed for these threshold data.*

We have also changed the following lines in results section to reflect this:

*Line 289-293: For computer based tests, there was a significant difference in stereo-thresholds between group (H(1,364)=54.38, p<0.0001; Kruskal-Wallis), and the overall test type (H(3,362)=56.76, p<0.0001; Kruskal-Wallis), However, there was not a significant effect of spatial frequency(H(3, 362)=1.98, p=0.58; Kruskal-Wallis).*

*Line 457-460 : For AIM, the thresholds for the 2 runs were not statistically different (z(96)=-0.336, p=0.73; Wilcoxon signed-rank test) and were averaged. The median of AIM stereo-thresholds were significantly higher (H(2,58)=18.12, p=0.0001; Kruskal-Wallis), than the stereo-thresholds from Randot or Titmus by a factor of 1.1-1.3 times.*

Usually, the F number is associated with two degrees of freedom not a single like F(5) and many more in the further text.

We have included the F number with two degrees of freedom wherever applicable.

Fig 5 Are the correlation values Pearson’s correlations? Also, correlation is denoted with the symbol r. In the last review, when I suggested reporting the r2 values, I meant only for those instances where the correlation value was higher and the regression line could be fit and could have reported the r2 values then. In the same context, Line 39 in the abstract should say correlation instead of agreement.

We used Kendall’s rank correlation and have noted that in the Statistical analysis section**.** We have added that in text and figure legend of Fig 5 and 10.

Following the reviewer’s comments in the first round, we report *r^2^* values in the revised manuscript. We use *r^2^* uniformly throughout the manuscript without assigning a criterion for the use of *r* or *r^2^*.

**Reviewer #2**: The authors have addressed all the comments of the reviewers. The manuscript is much better organized now. I have nothing further to add to this review.

We thank the reviewer for the appreciation.
